# Evidence that differentiation-inducing factor-1 controls chemotaxis and cell differentiation, at least in part, via mitochondria in *D. discoideum*

**DOI:** 10.1242/bio.021345

**Published:** 2017-06-15

**Authors:** Yuzuru Kubohara, Haruhisa Kikuchi, Van Hai Nguyen, Hidekazu Kuwayama, Yoshiteru Oshima

**Affiliations:** 1 Department of Molecular and Cellular Biology, Institute for Molecular and Cellular Regulation, Gunma University, Maebashi 371-8512, Japan; 2 Laboratory of Health and Life Science, Graduate School of Health and Sports Science, Juntendo University, Inzai, Chiba 270-1695, Japan; 3 Laboratory of Natural Product Chemistry, Graduate School of Pharmaceutical Sciences, Tohoku University, Sendai 980-8578, Japan; 4 Faculty of Life and Environmental Sciences, University of Tsukuba, Tsukuba 305-8572, Japan

**Keywords:** *Dictyostelium discoideum*, DIF-1, DIF-2, Mitochondria, Cell differentiation, Chemotaxis

## Abstract

Differentiation-inducing factor-1 [1-(3,5-dichloro-2,6-dihydroxy-4-methoxyphenyl)hexan-1-one (DIF-1)] is an important regulator of cell differentiation and chemotaxis in the development of the cellular slime mold *Dictyostelium discoideum*. However, the entire signaling pathways downstream of DIF-1 remain to be elucidated. To characterize DIF-1 and its potential receptor(s), we synthesized two fluorescent derivatives of DIF-1, boron-dipyrromethene (BODIPY)-conjugated DIF-1 (DIF-1-BODIPY) and nitrobenzoxadiazole (NBD)-conjugated DIF-1 (DIF-1-NBD), and investigated their biological activities and cellular localization. DIF-1-BODIPY (5 µM) and DIF-1 (2 nM) induced stalk cell differentiation in the DIF-deficient strain HM44 in the presence of cyclic adenosine monosphosphate (cAMP), whereas DIF-1-NBD (5 µM) hardly induced stalk cell differentiation under the same conditions. Microscopic analyses revealed that the biologically active derivative, DIF-1-BODIPY, was incorporated by stalk cells at late stages of differentiation and was localized to mitochondria. The mitochondrial uncouplers carbonyl cyanide *m*-chlorophenylhydrazone (CCCP), at 25–50 nM, and dinitrophenol (DNP), at 2.5–5 µM, induced partial stalk cell differentiation in HM44 in the presence of cAMP. DIF-1-BODIPY (1–2 µM) and DIF-1 (10 nM), as well as CCCP and DNP, suppressed chemotaxis in the wild-type strain Ax2 in shallow cAMP gradients. These results suggest that DIF-1-BODIPY and DIF-1 induce stalk cell differentiation and modulate chemotaxis, at least in part, by disturbing mitochondrial activity.

## Abstract (precis)

**Summary:** DIF-1 regulates *Dictyostelium* chemotaxis and differentiation. We synthesized DIF-1-BODIPY, a fluorescent DIF-1 derivative. DIF-1-BODIPY localized to mitochondria, suggesting that DIF-1 functions, at least in part, via mitochondria.

## INTRODUCTION

The vegetative amebae of the cellular slime mold *Dictyostelium discoideum* feed on bacteria. Starvation initiates morphogenesis: cells gather to form a slug-shaped multicellular aggregate and differentiate into two distinct types (prespore and prestalk), which eventually form a fruiting body consisting of spores and a multicellular stalk. Because of the simple pattern of its life cycle (cell differentiation and morphogenesis), *D. discoideum* is an excellent model in cell and developmental biology (Annesley and Fisher, 2009) (http://dictybase.org/).

**Fig. 1.**
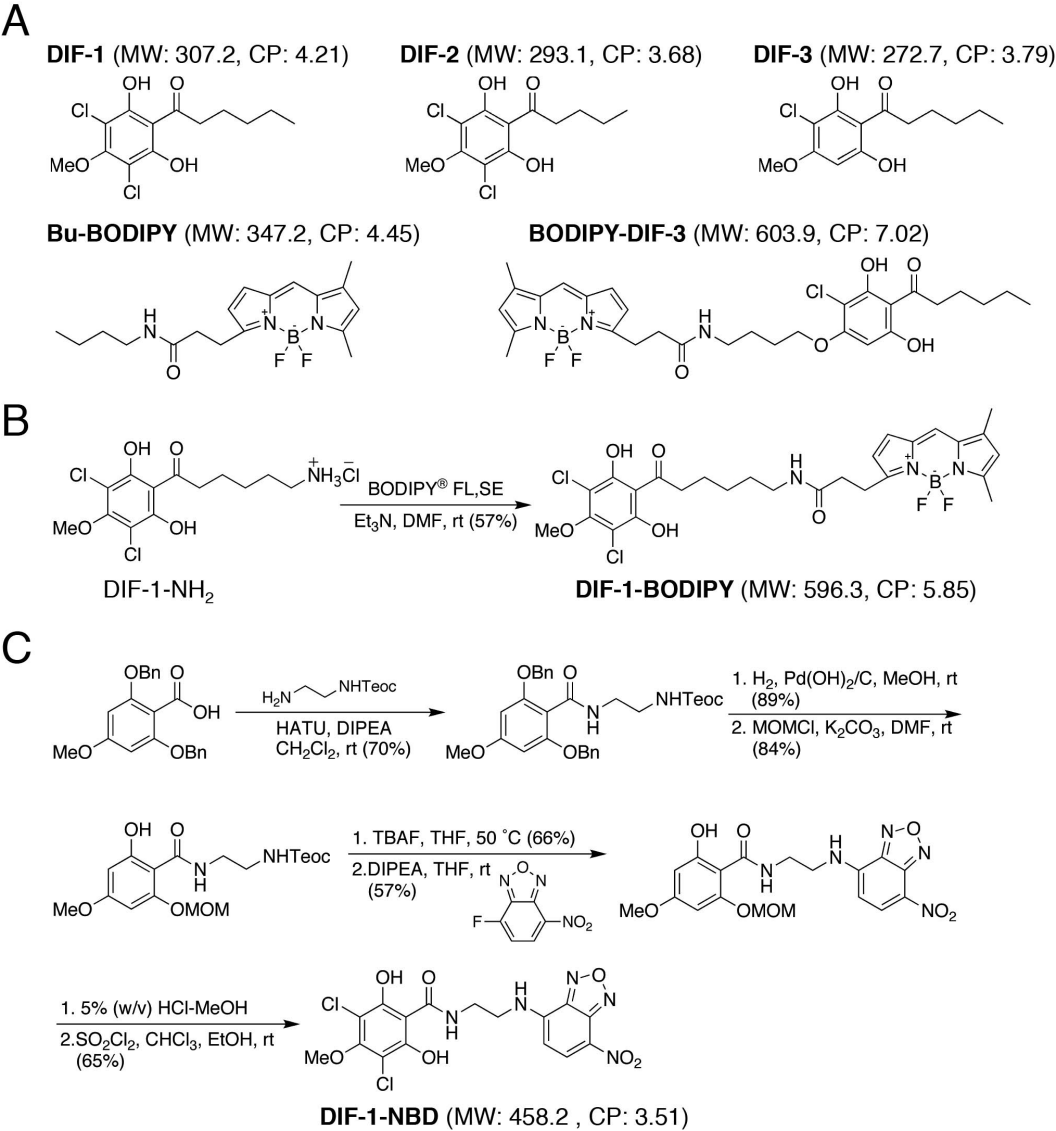
**Chemical structures of DIF-1 and related compounds.** (A) Chemical structures of DIFs, Bu-BODIPY and BODIPY-DIF-3. Molecular weight (MW) and CP for each compound are provided in parentheses. (B,C) Synthetic schemes of DIF-1-BODIPY and DIF-1-NBD. See Materials and Methods section for details.

Cyclic adenosine monosphosphate (cAMP) and the chlorinated polyketides differentiation-inducing factor-1 [1-(3,5-dichloro-2,6-dihydroxy-4-methoxyphenyl)hexan-1-one (DIF-1)] and differentiation-inducing factor-2 [1-(3,5-dichloro-2,6-dihydroxy-4-methoxyphenyl)pentan-1-one (DIF-2)] (Fig. 1A) play pivotal roles in the development of *D. discoideum.* While extracellular cAMP secreted by differentiating cells is essential for both prespore and prestalk cell differentiation, it also acts as a chemoattractant when cells gather to form the multicellular aggregate (Konijn et al., 1967; Bonner, 1970; Darmon et al., 1975; Kay, 1982). Initially, DIF-1 and DIF-2 were identified as inducers of stalk cell differentiation *in vitro* in the presence of cAMP (Town et al., 1976; Morris et al., 1987, 1988; Kay et al., 1989, 1999). The activity of DIF-1 is 2.5 times that of DIF-2 in *in vitro* assay with strains derived from V12M2, a wild-type strain (Kay et al., 1999; Masento et al., 1988). Differentiation-inducing factor-3 [1-(3-chloro-2,6-dihydroxy-4-methoxyphenyl)hexan-1-one (DIF-3)] (Fig. 1A) is the first metabolite produced during the degradation of DIF-1 and has virtually no activity in the induction of stalk cell differentiation in *D. discoideum* (Morris et al., 1988; Kay et al., 1989).

DIF-1 might function, at least in part, via increases in cytosolic calcium or proton concentrations (Kubohara and Okamoto, 1994; Schaap et al., 1996; Azhar et al., 1997; Kubohara et al., 2007; Lam et al., 2008). Several transcription factors, such as the basic-leucine zipper transcription factors, DimA and DimB, are involved in DIF-1 signaling (Thompson et al., 2004; Huang et al., 2006; Zhukovskaya et al., 2006; Keller and Thompson, 2008). In shallow cAMP gradients, DIF-1 inhibits chemotaxis via the phosphodiesterase GbpB, whereas DIF-2 stimulates chemotaxis via the phosphodiesterase RegA (Kuwayama and Kubohara, 2009; Kuwayama et al., 2011). The mechanisms by which DIFs modulate chemotaxis differ, at least in part, from those they use to induce stalk cell differentiation (Kuwayama and Kubohara, 2009, 2016; Kuwayama et al., 2011). Despite the importance of DIF-1 and DIF-2 in *D. discoideum* development, the entire signaling pathways they activate, including receptors, remain to be identified.

To elucidate the mechanisms underlying the effects of DIF-1 (and possibly DIF-2), we synthesized two fluorescent derivatives of DIF-1, boron-dipyrromethene (BODIPY)-conjugated DIF-1 (DIF-1-BODIPY) and nitrobenzoxadiazole (NBD)-conjugated DIF-1 (DIF-1-NBD) (Fig. 1B,C), and investigated their localization and function in *D. discoideum* cells. We show that DIF-1-BODIPY, but not DIF-1-NBD, is bioactive and appears to function similarly to DIF-1: this derivative induces stalk cell formation *in vitro* in the presence of cAMP in HM44 (a DIF-deficient strain) (Kopachik et al., 1983) and suppresses chemotaxis of cells of the wild-type strain Ax2 in shallow cAMP gradients. We also show that DIF-1-BODIPY is undetectable inside the cells during an early stage of development but is localized to intracellular organelles, mainly mitochondria, during a later developmental stage. We examined the effects of DIF-1, DIF-1-BODIPY, and the mitochondrial uncouplers dinitrophenol (DNP) and carbonyl cyanide *m*-chlorophenylhydrazone (CCCP), and the results suggest that DIF-1 (and DIF-1-BODIPY) induces stalk cell differentiation and modulates chemotaxis, at least in part, via mitochondria.

## RESULTS

### Synthesis of fluorescent derivatives of DIF-1 and assay of stalk cell induction

**Fig. 2.**
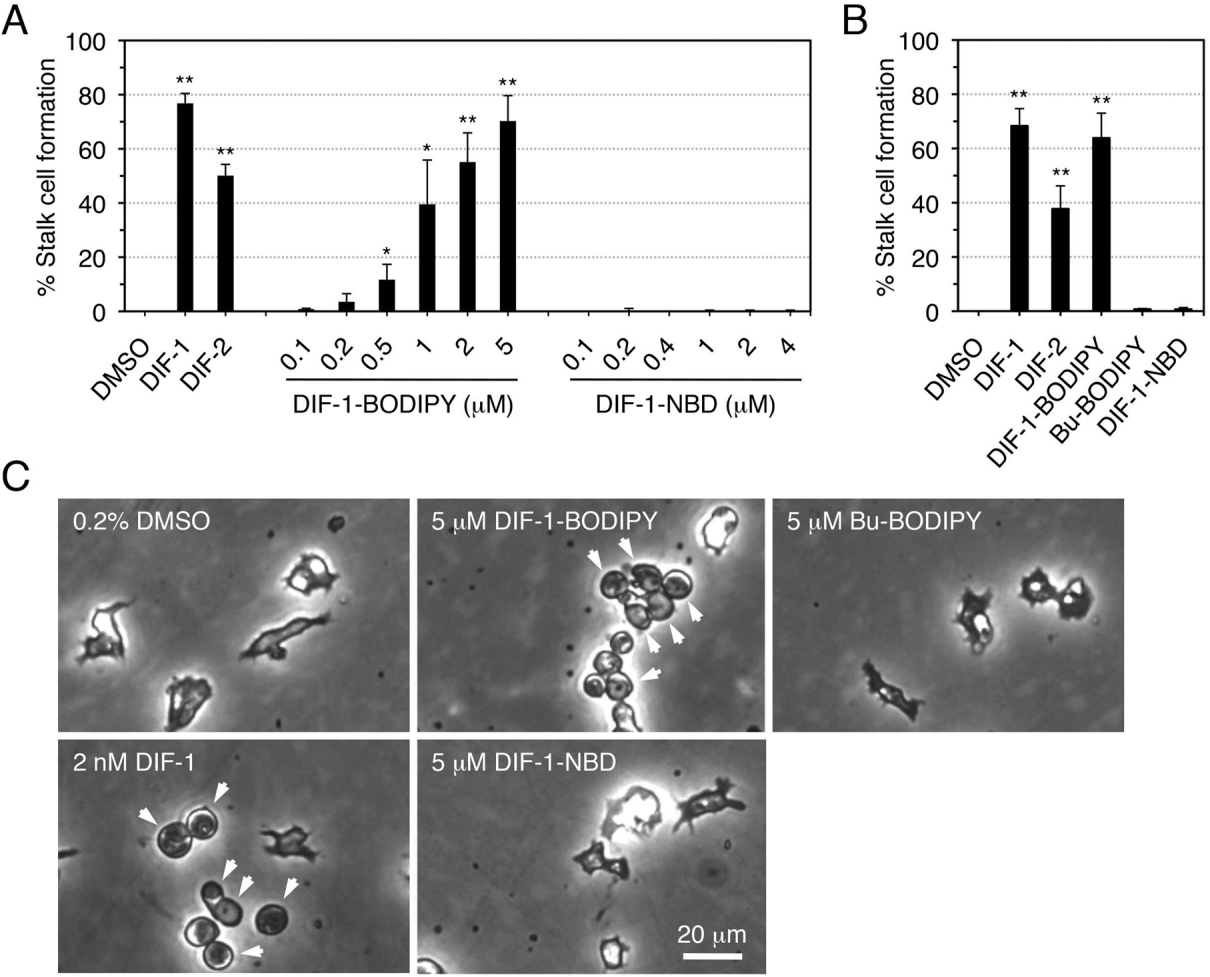
**Stalk-cell-inducing activities of DIF-1 and related compounds in HM44 cells.** (A) Cells were incubated *in vitro* for 48 h with 5 mM cAMP in the presence of 0.2% DMSO, 2 nM DIF-1 or DIF-2, or the indicated concentrations of DIF-1-BODIPY or DIF-1-NBD, and the stalk cell population was assessed by phase-contrast microscopy. (B) Cells were incubated *in vitro* for 48 h with 5 mM cAMP in the presence of 0.2% DMSO, 2 nM DIF-1 or DIF-2, or 5 µM DIF-1-BODIPY, Bu-BODIPY or DIF-1-NBD, and the stalk cell population was assessed by using phase-contrast microscopy. Data are the mean±s.d. of three independent experiments. *P<0.05; **P<0.01 (by one-tailed Welch's *t*-test). (C) Representative photos of the cells in (B); arrowheads indicate stalk cells with a vacuole.

The synthetic schemes of DIF-1-BODIPY and DIF-1-NBD are shown in Fig. 1B,C. We also synthesized the control compound butyl-BODIPY (Bu-BODIPY) (Kubohara et al., 2013). The effects of DIF-1, DIF-2, and the fluorescent compounds on *in vitro* stalk cell differentiation in the DIF-deficient strain HM44 are shown in Fig. 2. Even in the presence of cAMP, HM44 cells cannot differentiate into stalk cells *in vitro* unless exogenous DIF is supplied; therefore, HM44 cells are suitable for the assay of stalk cell induction by DIF-like molecules (Kopachik et al., 1983; Kubohara et al., 1993; Kubohara and Okamoto, 1994). As expected, DIF-1 or DIF-2 (2 nM) induced stalk cell formation in HM44 in the presence of cAMP; DIF-1-BODIPY (0.1–5 µM) dose-dependently induced stalk cell formation in up to 60%–80% of the cells under the same conditions (Fig. 2). By contrast, neither Bu-BODIPY (5 µM) nor DIF-1-NBD (0.1–5 µM) induced any stalk cell formation (Fig. 2).

### Cellular localization of DIF-1-BODIPY during *in vitro* stalk cell differentiation

**Fig. 3.**
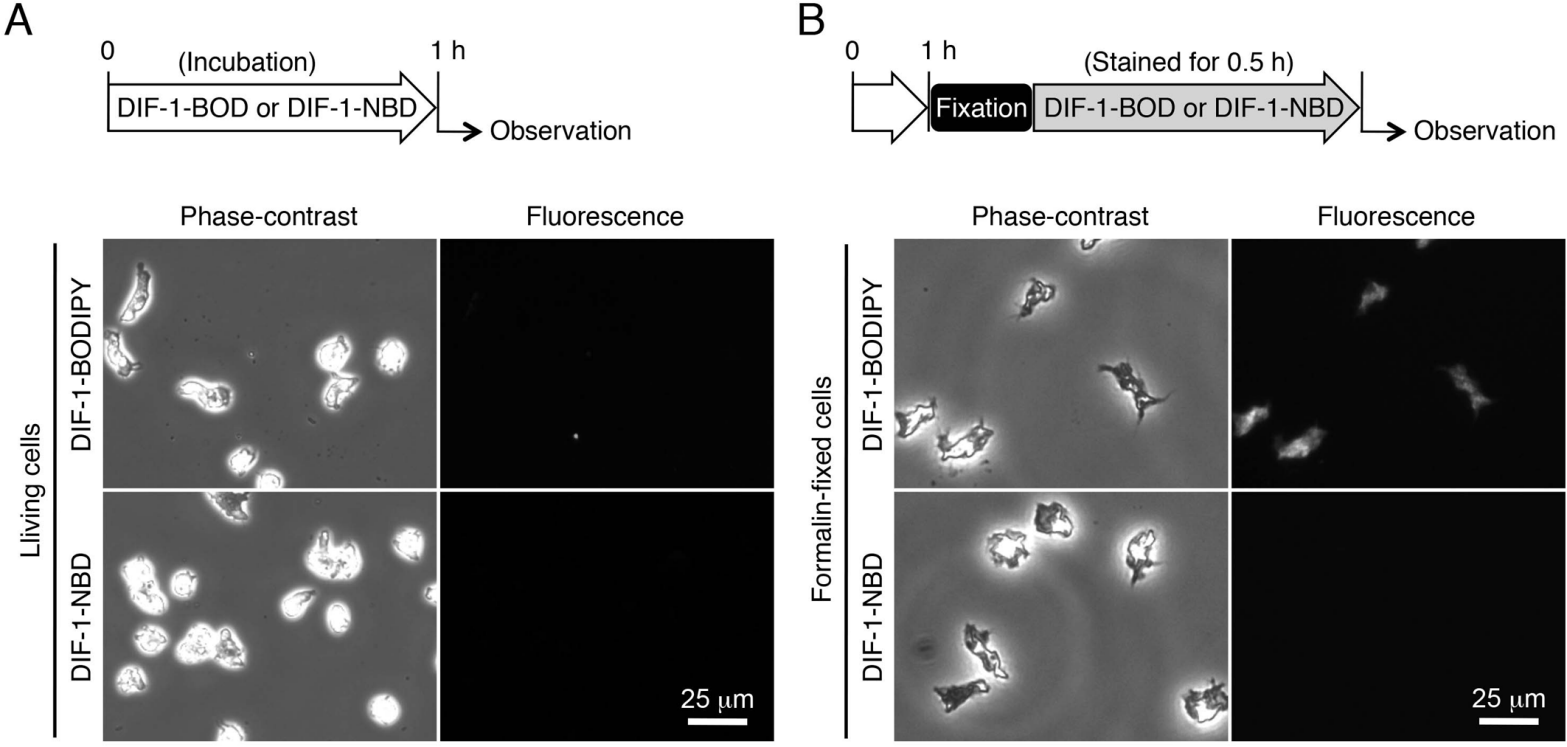
**Localization of DIF-1-BODIPY and DIF-1-NBD in living and formalin-fixed HM44 cells.** (A) Cells were incubated *in vitro* for 1 h with 5 µM DIF-1-BODIPY or DIF-1-NBD. (B) Cells were incubated *in vitro* for 1 h with no additives, fixed with formalin, and stained for 0.5 h with 5 µM DIF-1-BODIPY or DIF-1-NBD. Cells were washed free of the additives and observed under phase-contrast and fluorescence microscopes. Scheme of the experiment is indicated above each panel.

We next compared the cellular localization of DIF-1-BODIPY and DIF-1-NBD in HM44 cells. After 1-h starvation (incubation), cells were ameboid and were hardly stained with DIF-1-BODIPY or DIF-1-NBD (Fig. 3A), whereas cells fixed with formalin after starvation were stained well with the bioactive derivative DIF-1-BODIPY, but not with the nonbioactive derivative DIF-1-NBD (Fig. 3B).

**Fig. 4.**
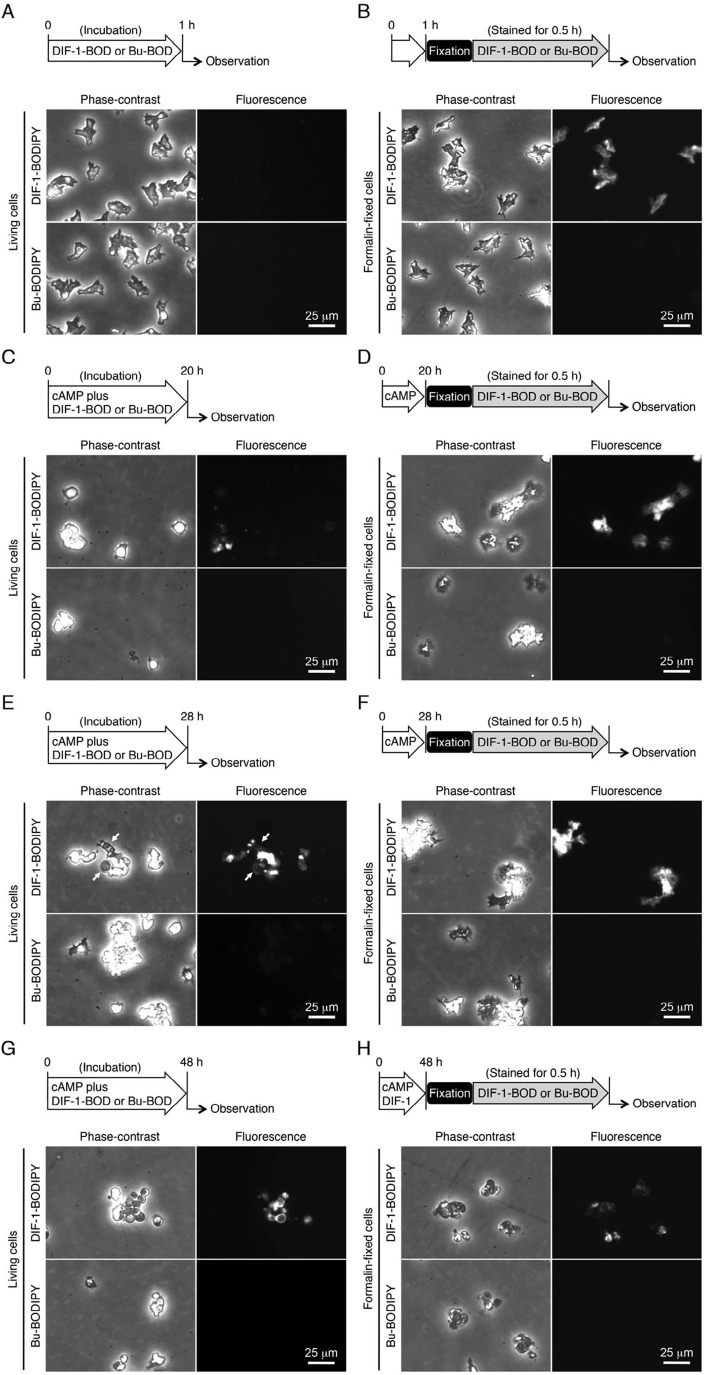
**Localization of DIF-1-BODIPY and Bu-BODIPY in living and formalin-fixed HM44 cells.** (A,B) Localization of DIF-1-BODIPY and Bu-BODIPY in undifferentiated cells. (A) Cells were incubated *in vitro* for 1 h with 5 µM DIF-1-BODIPY (DIF-1-BOD) or Bu-BODIPY (Bu-BOD). (B) Cells were incubated *in vitro* for 1 h without the additives, fixed with formalin, and stained for 0.5 h with 5 µM DIF-1-BODIPY or Bu-BODIPY. Cells were washed free of the additives and observed under phase-contrast and fluorescence microscopes. (C–F) Localization of DIF-1-BODIPY and Bu-BODIPY in differentiating cells. (C,E) Cells were incubated *in vitro* for (C) 20 h or (E) 28 h with 5 mM cAMP in the presence of 5 µM DIF-1-BODIPY or Bu-BODIPY. (D,F) Cells were incubated for the same time periods with 5 mM cAMP, fixed with formalin, and stained for 0.5 h with 5 µM DIF-1-BODIPY or Bu-BODIPY. Cells were washed and observed under phase-contrast and fluorescence microscopes. (G,H) Localization of DIF-1-BODIPY and Bu-BODIPY in stalk cells. (G) Cells were incubated *in vitro* for 48 h with 5 mM cAMP in the presence of 5 µM DIF-1-BODIPY or Bu-BODIPY. (H) Cells were incubated for the same time periods with 5 mM cAMP, fixed with formalin, and stained for 0.5 h with 5 µM DIF-1-BODIPY or Bu-BODIPY. Cells were washed and observed under phase-contrast and fluorescence microscopes. Scheme of the experiment is indicated above each panel.

We then compared cellular localization of DIF-1-BODIPY and the nonbioactive control compound Bu-BODIPY during *in vitro* differentiation of HM44 cells. After 1-h starvation (incubation), cells were hardly stained with DIF-1-BODIPY (Fig. 4A). After 20-h incubation with cAMP and DIF-1-BODIPY, cells were still ameboid; some of them had formed aggregates, in which some cells were stained with DIF-1-BODIPY, and there was heterogeneity among the cells (Fig. 4C). At 28 h, cells had begun to differentiate into stalk cells; one or more autophagic vacuoles had formed in each cell, each cell had formed a cell wall, and many cells were stained with DIF-1-BODIPY to a variable extent (Fig. 4E). At 48 h, most cells had differentiated into stalk cells and were stained with DIF-1-BODIPY; the signal was stronger in cytoplasmic regions than in autophagic vacuoles (Fig. 4G). However, cells fixed with formalin were stained with DIF-1-BODIPY at each time point (Fig. 4B,D,F,G). These observations suggest that DIF-1-BODIPY (and possibly DIF-1) is unable to penetrate into the cells or is pumped out from the cells during the early phase, but not during later phases, of cell differentiation. By contrast, Bu-BODIPY neither induced stalk cell formation nor was detected in the cells at any time point, even if they were fixed with formalin (Fig. 4). Taken together, these results indicate that DIF-1-BODIPY can be used to probe cellular uptake and localization of DIF-1.

### Target organelle of DIF-1-BODIPY

**Fig. 5.**
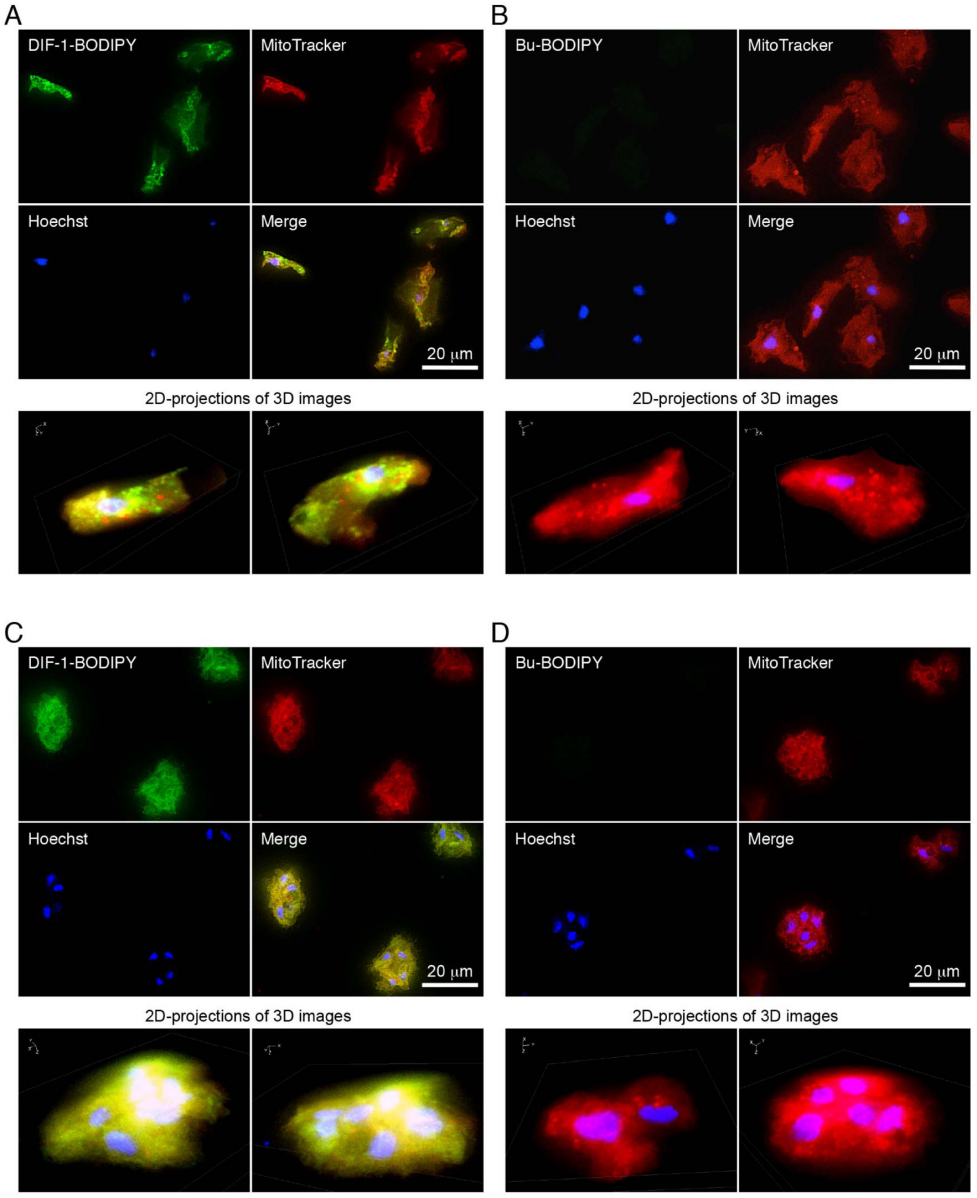
**Multi-color imaging of formalin-fixed HM44 cells.** (A,B) Cells were incubated *in vitro* for 1 h with MitoTracker (0.2 µM) and fixed with formalin. Cells were then stained for 0.5 h with Hoechst (1 µg ml^−1^) and (A) 5 µM DIF-1-BODIPY or (B) Bu-BODIPY, then washed and observed by using high-magnification fluorescence microscopy. (C,D) Cells were incubated *in vitro* for 20 h with 5 mM cAMP, further incubated for 1 h with MitoTracker (0.2 µM), and fixed with formalin. Cells were then stained for 0.5 h with Hoechst and (C) 5 µM DIF-1-BODIPY or (D) Bu-BODIPY, then washed and observed as above. Three-dimensional images were constructed from z-stacked two-dimensional (2D) images; two representative 2D projections of the 3D images are shown. Nonlinear adjustment was performed on 3D images to obtain clear high-contrast images. Note that DIF-1-BODIPY and MitoTracker co-localized to mitochondria.

It is noteworthy that DIF-1, DIF-3 and their derivatives possess anti-tumor activities (Asahi et al., 1995; Kubohara, 1997, 1999; Gokan et al., 2005), and that DIF-3 derivatives are more active than DIF-1 derivatives in suppressing tumor cell growth (Gokan et al., 2005; Kubohara, 1999). We have shown that the fluorescent derivative BODIPY-DIF-3 (Fig. 1A) localizes to mitochondria in mammalian cell lines (Kubohara et al., 2013, 2014). We thus compared localization of DIF-1-BODIPY and MitoTracker (a probe for mitochondria) in HM44 cells (Fig. 5). DIF-1-BODIPY co-localized to mitochondria stained with MitoTracker in formalin-fixed cells that had been starved for 1 h (Fig. 5A) or incubated for 21 h with cAMP; in the latter case, most cells had formed small aggregates (Fig. 5C). By contrast, Bu-BODIPY did not stain any organelles in formalin-fixed cells (Fig. 5B,D). These results strongly suggest that DIF-1-BODIPY (and possibly DIF-1) might function, at least in part, by affecting mitochondrial activity in *D. discoideum*.

### Effects of CCCP and DNP on stalk cell differentiation

**Fig. 6.**
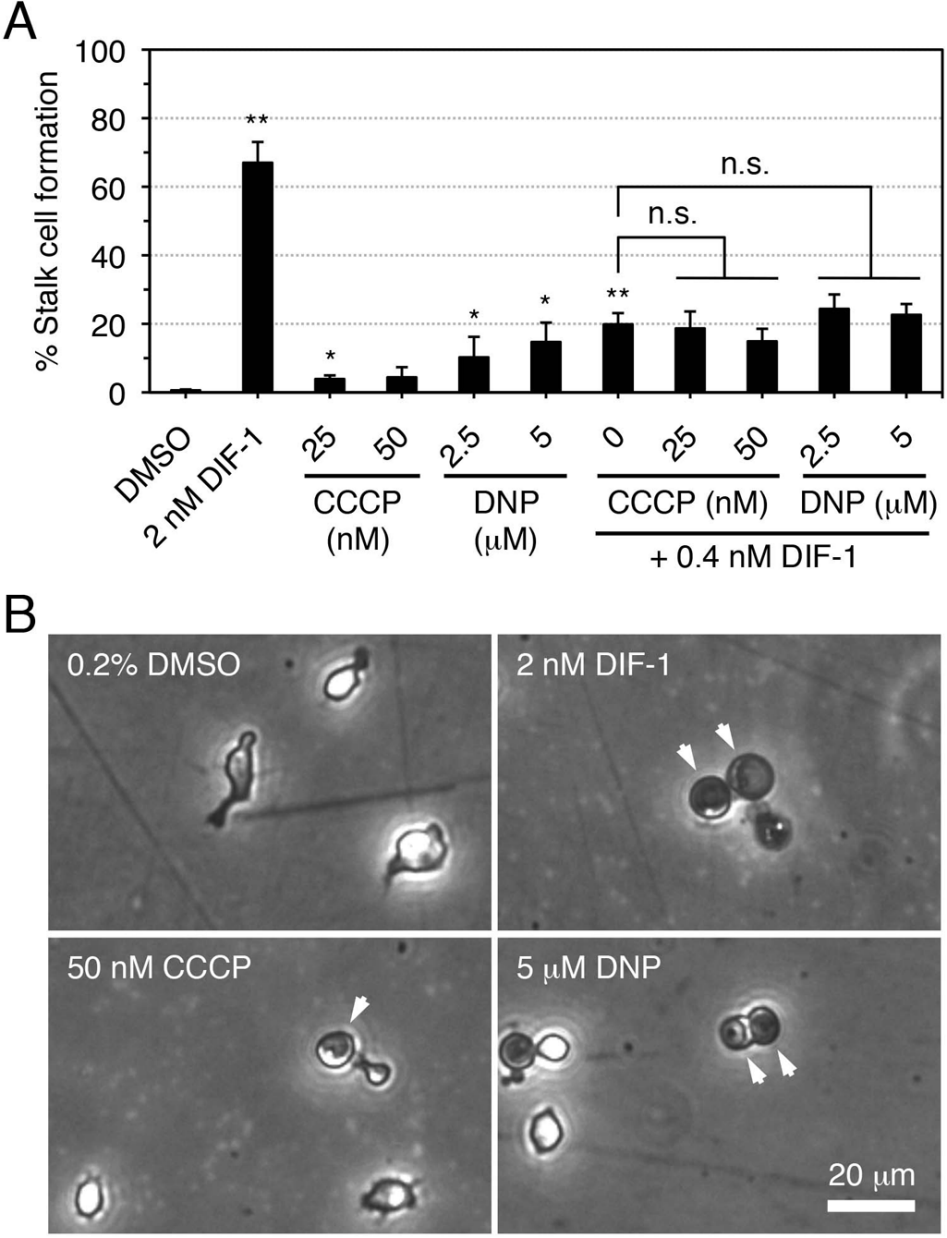
**Effects of CCCP and DNP on stalk cell formation in HM44.** (A) Cells were incubated *in vitro* for 48 h with 5 mM cAMP in the presence of 0.2% DMSO or the indicated concentrations of CCCP, DNP, and/or DIF-1, and the stalk cell population was assessed by using phase-contrast microscopy. Data are the mean±s.d. of three independent experiments. *P<0.05; **P<0.01 versus DMSO control; n.s., not significant (by one-tailed Welch's *t*-test). (B) Representative images of cells incubated for 48 h with the indicated compounds. Arrowheads indicate stalk cells.

We have recently shown that DIF-1 and its derivatives act as mitochondrial uncouplers in mammalian cells (Kubohara et al., 2013, 2015). To determine whether DIF-1 (and DIF-1-BODIPY) induces stalk cell differentiation by affecting mitochondria in *D. discoideum*, we examined the effects of CCCP and DNP on stalk cell formation in HM44. As shown in Fig. 6, CCCP (25–50 nM) or DNP (2.5–5 µM) weakly but significantly induced stalk cell formation in the presence of cAMP; at higher concentrations, both uncouplers were toxic to the cells (data not shown). The stalk-inducing activities of CCCP and DNP did not exceed ∼10% and ∼20%, respectively (Fig. 6A); neither CCCP nor DNP showed additive effects with DIF-1 at a low concentration (0.4 nM). These results suggest that DIF-1 induces stalk cell differentiation partly by uncoupling mitochondrial activity but also via another pathway.

### Effects of DIF-1-BODIPY on chemotactic cell movement

**Fig. 7.**
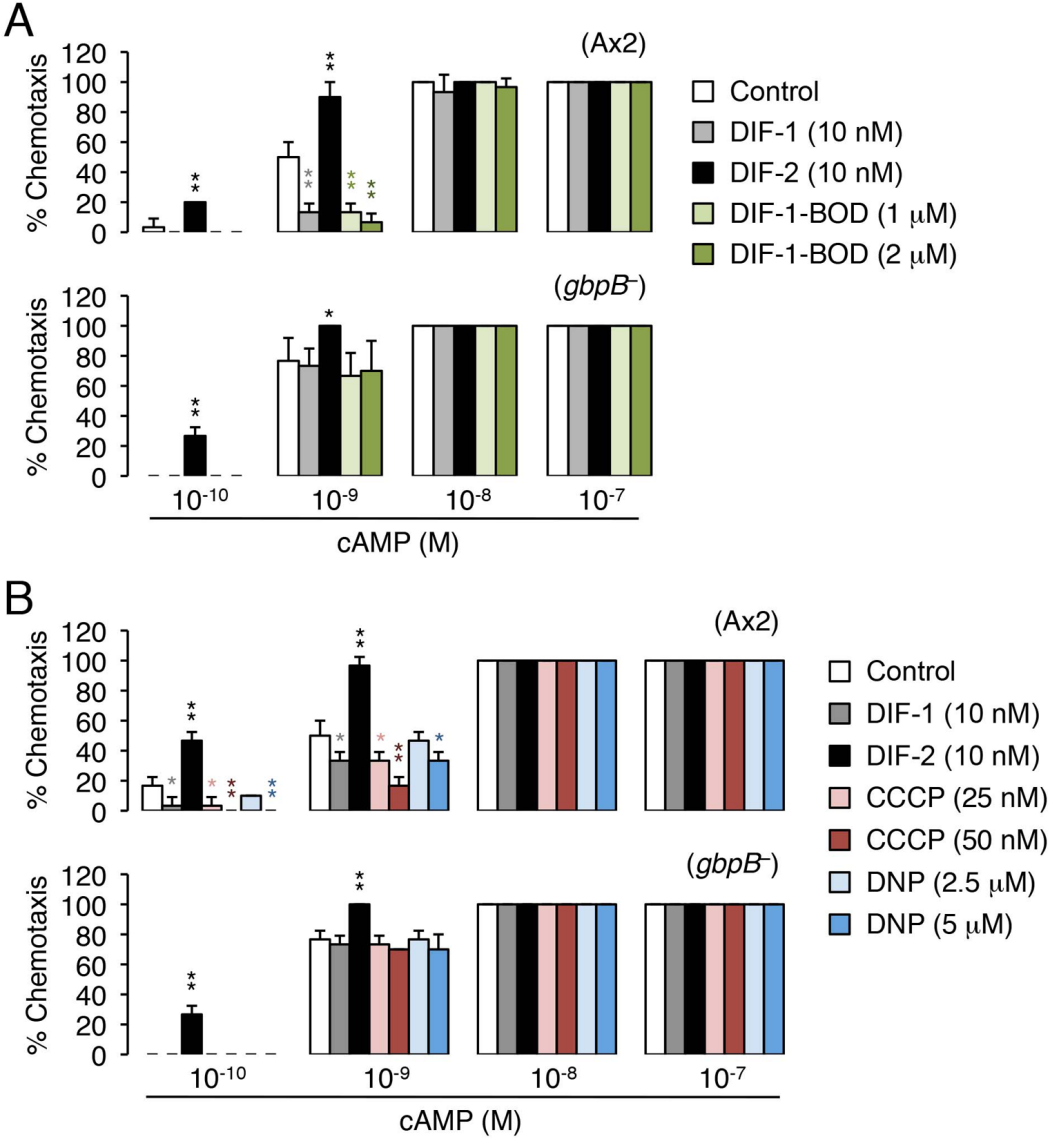
**Effects of DIF-1-BODIPY, CCCP, and DNP on chemotaxis toward cAMP.** Ax2 and *gbpB*^–^ cells were starved for 6 h, and cell droplets were placed on phosphate-buffered agar containing 3 mM caffeine (control) plus (A) DIF-1, DIF-2, or DIF-1-BODIPY (DIF-1-BOD) or (B) DIF-1, DIF-2, CCCP, or DNP. Cells were assayed for chemotaxis at the indicated concentrations of cAMP (10 cell droplets per concentration per plate were examined). Data are the mean±s.d. for triplicate sample plates. *P<0.05; **P<0.01 versus control cells (by one-tailed Welch's *t*-test).

To verify whether DIF-1-BODIPY inhibits chemotaxis (similar to DIF-1) or stimulates chemotaxis (similar to DIF-2), we examined its effects on chemotactic movement of Ax2 cells toward cAMP (Fig. 7A). In shallow cAMP gradients, chemotaxis was suppressed by 10 nM DIF-1 and promoted by 10 nM DIF-2, as previously described (Kuwayama and Kubohara, 2009). Similar to DIF-1, DIF-1-BODIPY (1–2 µM) suppressed chemotaxis (Fig. 7A).

To confirm that DIF-1-BODIPY can functionally mimic DIF-1, we next compared their effects on chemotaxis in *gbpB*^–^ cells. Neither DIF-1 (10 nM) nor DIF-1-BODIPY (1–2 µM) affected chemotaxis in shallow cAMP gradients, whereas DIF-2 (used as a positive control) significantly promoted chemotaxis under the same conditions (Fig. 7A), indicating that DIF-1-BODIPY and DIF-1 exert their effects via a GbpB-dependent pathway (Kuwayama and Kubohara, 2009). Thus, DIF-1-BODIPY can functionally mimic DIF-1 in the regulation of chemotaxis.

### Effects of CCCP and DNP on chemotactic cell movement

To demonstrate that DIF-1 might function by disturbing mitochondrial activity, we examined the effects of CCCP and DNP on chemotactic cell movement toward cAMP (Fig. 7B). As expected, CCCP (25–50 nM), DNP (5 µM) and DIF-1 (10 nM) significantly suppressed chemotaxis of Ax2 cells in shallow cAMP gradients but hardly affected chemotaxis of *gbpB*^–^ cells (Fig. 7B). These results indicate that all three compounds suppress chemotaxis via a GbpB-dependent pathway and that DIF-1 (and possibly DIF-1-BODIPY) might suppress chemotaxis in shallow cAMP gradients by uncoupling mitochondrial activity.

### Localization of DIF-1-BODIPY in aggregating Ax2 cells

Finally, we localized DIF-1-BODIPY in aggregating (chemotacting) Ax2 cells under submerged conditions without exogenous cAMP; under these conditions, cells formed streaming aggregates (Fig. S1). After 3-h incubation, we still observed single ameboid cells; living cells were not stained with DIF-1-BODIPY, although formalin-fixed cells were clearly stained (Fig. S1A). At 15 h, cells formed aggregates, in which a small fraction of the cells was clearly stained with DIF-1-BODIPY; formalin-fixed cells were strongly stained (Fig. S1B).

## DISCUSSION

### Biological activities of DIF-1-BODIPY

In this study, we designed and synthesized the fluorescent DIF derivative DIF-1-BODIPY (Fig. 1B) and found that DIF-1-BODIPY (0.1–5 µM) induced stalk cell differentiation in the presence of cAMP in the DIF-deficient strain HM44 (Fig. 2). DIF-1-BODIPY (1–2 µM) also suppressed chemotaxis in shallow cAMP gradients in Ax2 cells (Fig. 7). Although we do not exclude the possibility that DIF-1-BODIPY at several micromolars might affect cellular functions nonspecifically, the present results indicate that DIF-1-BODIPY can mimic the effects of DIF-1 in *D. discoideum*.

### Subcellular localization of DIF-1-BODIPY to mitochondria

**Fig. 8.**
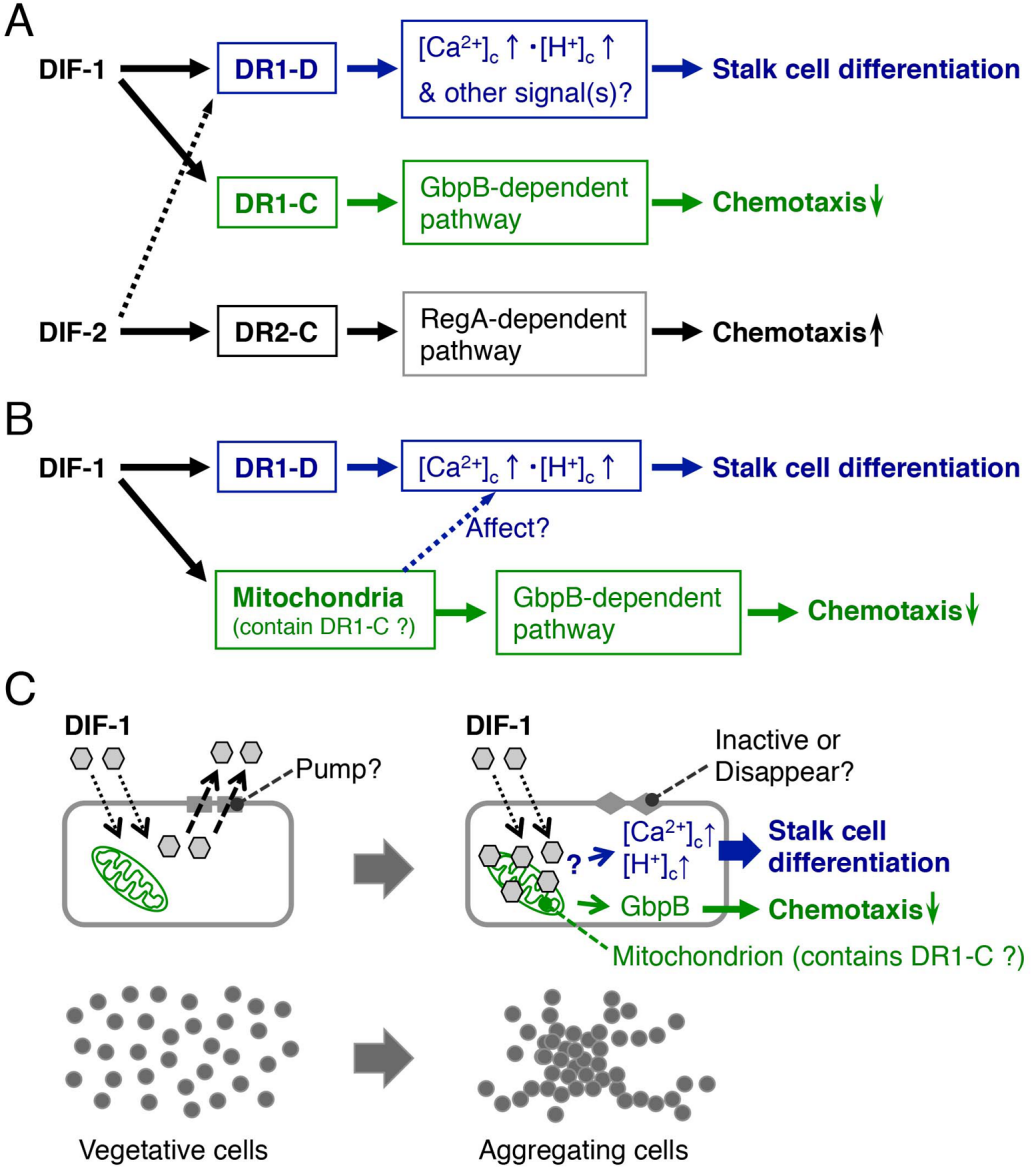
**Proposed scheme for DIF signaling pathways in *D. discoideum*.** (A) Hypothetical receptors for DIF-1 and DIF-2 control cell differentiation and chemotaxis. We assume that DIF-1 has two receptors: DR1-D (DR-1 in Kuwayama et al., 2011) and DR1-C (DR-2 in Kuwayama et al., 2011); DIF-2 has one receptor, DR2-C (DR-3 in Kuwayama et al., 2011). We hypothesize that (1) DIF-1 induces stalk cell differentiation via DR1-D (DIF-1 receptor responsible for induction of cell differentiation) and an increase in cytosolic calcium and proton concentrations (Kubohara and Okamoto, 1994; Kubohara et al., 2007); (2) DIF-1 suppresses chemotactic cell movement in shallow cAMP gradients via DR1-C (DIF-1 receptor responsible for modulation of chemotaxis) and the GbpB-dependent pathway; and (3) DIF-2 promotes chemotactic cell movement in shallow cAMP gradients via DR2-C (DIF-2 receptor responsible for modulation of chemotaxis) and the *Dictyostelium* histidine kinase C (DhkC)-RegA-dependent pathway (Kuwayama and Kubohara, 2016, 2009; Kuwayama et al., 2011). DhkC might function as DR2-C (Kuwayama and Kubohara, 2016). DIF-2 would also induce stalk cell differentiation via DR1-D (dotted arrow) due to its structural similarity to DIF-1. (B) Newly proposed scheme for chemotaxis modulation by DIF-1. DIF-1 localizes to mitochondria and suppresses chemotaxis in shallow cAMP gradients. Because CCCP and DNP, similar to DIF-1, suppress chemotaxis in shallow cAMP gradients, DIF-1 might suppress chemotactic cell movement via mitochondria and the GbpB-dependent pathway; DR1-C might reside in mitochondria. (C) Cellular localization of DIF-1 during early cell differentiation. In vegetative cells, DIF-1 penetrates the cell membrane but is continuously pumped out of the cells; in differentiating (aggregating) cells, DIF-1 is retained in some cells because of inactivation or disappearance of the pump. It is localized to mitochondria and promotes stalk cell differentiation.

The hydrophobic indices of DIF-1 [ClogP (CP), 4.21] and DIF-1-BODIPY (CP, 5.85) (Fig. 1A,B) indicate that both compounds are likely to penetrate the cell membrane. However, we found that DIF-1-BODIPY was absent in cells at early stages of development but gradually penetrated into or was taken up by cells differentiating to stalk cells (Fig. 4). DIF-1-BODIPY localized to mitochondria (Fig. 5). During early, but not late, stages of development, DIF-1-BODIPY (and thus DIF-1) might be pumped out of cells (Fig. 8). The molecular size of DIF-1-BODIPY is larger than that of DIF-1 and the hydrophobic indices of the two compounds are different (Fig. 1A,B). However, because Bu-BODIPY was not detected in any organelles (Figs 4 and 5), the mitochondrial localization of DIF-1-BODIPY was not caused by the BODIPY moiety but likely reflects localization of DIF-1.

DIFs and their derivatives possess anti-tumor activities when tested on mammalian tumor cells, and derivatives of DIF-3 are more potent anti-tumor agents than those of DIF-1 (Asahi et al., 1995; Gokan et al., 2005; Kubohara, 1999; Takahashi-Yanaga et al., 2003; Akaishi et al., 2004; Kubohara et al., 2015; Oladimeji et al., 2015). The fluorescent DIF-3 derivative BODIPY-DIF-3 penetrates the cell membrane, localizes to mitochondria, and suppresses cell growth in some of the tumor cell lines tested (Kubohara et al., 2013, 2015). Bioactive DIF derivatives and the mitochondrial uncoupler CCCP promote oxygen consumption in mitochondria isolated from mouse liver; the anti-tumor activity of DIF derivatives might result, at least in part, from their uncoupling activity in the mitochondria of tumor cells (Kubohara et al., 2013, 2015). In *D. discoideum*, DIF-1 can disturb mitochondrial membrane potential and respiration, suggesting that it might function as an uncoupler (Shaulsky and Loomis, 1995), although the effective concentration range of DIF-1 (0.1–1 µM) rather exceeded its putative physiological concentrations (at most ∼0.1 µM; Kay, 1998). However, DIF-1 at 0.1 µM was later shown to affect mitochondrial membrane potential in Ax2 cells (Arnoult et al., 2001) and to promote mitochondrial oxygen consumption and induce ATP depletion in an autophagy mutant strain (Laporte et al., 2007; Giusti et al., 2009), suggesting that it can act as an uncoupler at physiological concentrations. In the present study, we have shown that the mitochondrial uncouplers CCCP and DNP induce partial stalk differentiation of HM44 cells (Fig. 6) and that the uncouplers and DIF-1 suppress chemotaxis in Ax2 cells in shallow cAMP gradients (Fig. 7B). Taken together, these data suggest that DIF-1 might function, at least in part, via mitochondria (possibly as an uncoupler) in *D. discoideum*. Unexpectedly, however, neither CCCP (25–50 nM) nor DNP (2.5–5 µM) showed an additive effect with 0.4 nM DIF-1 on stalk cell formation (Fig. 6). Although the cause of the absence of such effect is unknown, DIF-1 might function via multiple signaling cascades, only one of which may be mimicked by CCCP and DNP; DIF-1 at 0.4 nM might be sufficient to saturate this pathway. Alternatively, as CCCP >50 nM and DNP >5 µM were toxic to the cells (data not shown), their toxicity might cancel their stalk-inducing activity in the presence of DIF-1.

### Biological activity and cellular localization of DIF-1-NBD

In the present study, we have also synthesized DIF-1-NBD, a fluorescent amide derivative of DIF-1 (Fig. 1C). DIF-1-NBD was expected to be a good probe for DIF-1 because some amide derivatives of DIF-1 are excellent inducers of stalk cell differentiation in HM44 cells (Kikuchi et al., 2008), and also because the molecular size of DIF-1-NBD is much smaller than that of DIF-1-BODIPY and its CP value (3.51) suggests that DIF-1-NBD can penetrate the cell membrane (Fig. 1A). Unfortunately, however, DIF-1-NBD (5 µM) neither induced stalk cell differentiation (Fig. 2) nor appeared to localize to any parts of HM44 cells (Fig. 3). Although we cannot exclude that the localization of DIF-1-BODIPY in mitochondria reflects its non-specific binding because of its high concentration, the absence of cell staining or activity of DIF-1-NBD (5 µM) and another non-bioactive compound Bu-BODIPY (5 µM) (Figs 2–4) indicates that it is likely that the biological activities and cellular localization of DIF-1-BODIPY (5 µM) reflect those of DIF-1 at nanomolar concentrations.

### Proposed scheme for DIF-1 function

The functions of DIF-1 have been analyzed in many studies (Kubohara and Okamoto, 1994; Schaap et al., 1996; Azhar et al., 1997; Thompson et al., 2004; Huang et al., 2006; Zhukovskaya et al., 2006; Kubohara et al., 2007; Lam et al., 2008; Keller and Thompson, 2008; Luciani et al., 2009; Giusti et al., 2010). As we have shown here the possible involvement of mitochondria in the effects of DIF-1, we mainly discuss the relationship between DIF signaling and mitochondria. We assume that DR1-D (putative DIF-1 receptor responsible for the induction of cell differentiation) (Fig. 8A) might mediate the induction of stalk cell differentiation by DIF-1 (at least in part via increases in intracellular calcium and/or proton concentrations) (Kubohara and Okamoto, 1994; Schaap et al., 1996; Azhar et al., 1997; Kubohara et al., 2007; Lam et al., 2008). In shallow cAMP gradients, DIF-1 suppresses chemotaxis via a GbpB-dependent pathway, whereas DIF-2 promotes chemotaxis via a RegA-dependent pathway (Kuwayama and Kubohara, 2009; Kuwayama et al., 2011); we assume here that DIF-1 functions via DR1-C (putative DIF-1 receptor responsible for modulation of chemotaxis) and that DIF-2 functions via DR2-C (putative DIF-2 receptor responsible for modulation of chemotaxis) (Fig. 8A). DIF-1 is likely to modulate chemotaxis by interfering with mitochondrial activity; if so, mitochondria might be the target organelles of DIF-1 that contain DR1-C (Fig. 8B). CCCP and DNP induce partial stalk cell differentiation (Fig. 6) and mitochondria affect intracellular calcium and proton concentrations (Swietach et al., 2013; de Marchi et al., 2014); therefore, DIF-1 might induce stalk cell formation, at least in part, by disturbing (uncoupling) mitochondrial activity (Fig. 8B,C).

## MATERIALS AND METHODS

### Cell lines and cell culture

The *D. discoideum* DIF-deficient strain HM44 (Kopachik et al., 1983) was used for *in vitro* stalk cell induction assay. The axenic strain Ax2 and the *gbpB* null strain *gbpB*^–^ derived from Ax2 (Bosgraaf et al., 2002a,b; Goldberg et al., 2002) were used for chemotaxis assay. These strains were obtained from the National BioResource Project (NBRP Nenkin, Tsukuba, Japan). HM44 cells were grown in association with *Klebsiella aerogenes* on a modified SM agar plate (Inouye, 1988) at 21°C, whereas Ax2 and *gbpB*^–^ cells were grown axenically at 21°C in HL-5 liquid medium (Sussman, 1987). Growing cells were collected by centrifugation (500× ***g***, 3 min).

### Reagents

DIF-1, DIF-2, and Bu-BODIPY (Fig. 1A) were synthesized as described previously (Gokan et al., 2005; Kubohara et al., 2013); they were dissolved in ethanol or dimethyl sulfoxide (DMSO) and stored at −20°C. Amino derivative of DIF-1 [6-amino-1-(3,5-dichloro-2,6-dihydroxy-4-methoxyphenyl)hexan-1-one (DIF-1-NH_2_)] (Fig. 1B) was synthesized as described previously (Kubohara et al., 2010). MitoTracker Red CMXRos (referred to as MitoTracker) (Ex=579 nm, Em=599 nm) and BODIPY FL, SE (succinimidyl ester) (Ex=505 nm, Em=513 nm) were purchased from Invitrogen. Hoechst 33342 (Ex=352 nm, Em=461 nm) solution (1 mg ml^−1^ in H_2_O), CCCP, and DNP were obtained from Wako Pure Chemical Industries (Osaka, Japan). NBD-F (4-fluoro-7-nitro-2,1,3-benzoxadiazole) (Ex=470 nm, Em=530 nm) was from Tokyo Chemical Industry Co., Ltd. (Tokyo, Japan).

### Synthesis of DIF-1-BODIPY

As depicted in Fig. 1B, DIF-1-NH_2_ (1.3 mg, 3.5 µmol) and triethylamine (10 µl) were added to a solution of BODIPY FL, SE (2.1 mg, 5.3 µmol) in *N,N*-dimethylformamide (0.5 ml) at room temperature (*rt* in Fig. 1B) in the dark. The reaction mixture was stirred for 12 h and then diluted with 0.2 M hydrochloric acid (5 ml) and extracted with ethyl acetate (10 ml) three times. The residue was subjected to recycle preparative high-performance liquid chromotography (HPLC) on a JAIGEL-GS-310 column (φ 20 mm×500 mm) (Japan Analytical Industry Co., Ltd., Tokyo, Japan) in chloroform to give DIF-1-BODIPY (1.2 mg, 2.0 µmol). Analytical data for DIF-1-BODIPY: ^1^H nuclear magnetic resonance (NMR) [400 MHz, deuterated chloroform (CDCl_3_)] δ 10.75–10.92 (1H, br.s), 7.08 (1H, s), 6.88 (1H, d, *J*=4.0 Hz), 6.30 (1H, d, *J*=4.0 Hz), 6.11 (1H, s), 5.80–5.88 (1H, br.s), 3.98 (3H, s), 3.28 (2H, t, *J*=7.4 Hz), 3.23 (2H, q, *J*=6.9 Hz), 3.05 (2H, t, *J*=7.6 Hz), 2.66 (2H, t, *J*=7.4 Hz), 2.56 (3H, s), 2.24 (3H, s), 1.67 (2H, quint, *J*=7.4 Hz), 1.47 (2H, quint, *J*=7.4 Hz), 1.36 (2H, quint, *J*=7.4 Hz); high resolution fast-atom bombardment mass spectrometry (HRFABMS) *m/z* 577.1645 [M-F]^+^ (577.1640 calculated for C_27_H_30_N_3_O_5_B^35^Cl_2_F).

### Synthesis of DIF-1-NBD

DIF-1-NBD was synthesized as follows (Fig. 1C). To a solution of 2,6-bis(benzyloxy)-4-methoxybenzoic acid (Kikuchi et al., 2008) (100 mg, 0.274 mmol) in dichloromethane (8.0 ml), 2-(trimethylsilyl)ethyl 2-aminoethylcarbamate (61.3 mg, 0.300 mmol), *O*-(7-aza-1*H*-benzotriazol-1-yl)-*N*,*N*,*N′*,*N′*-tetramethyluronium hexafluorophosphate (HATU) (103 mg, 0.274 mmol) and *N*,*N*-diisopropylethylamine (71 µl, 0.407 mmol) were added at room temperature (*rt* in Fig. 1C). The reaction mixture was stirred for 1 h, poured into water and extracted with ethyl acetate three times. The combined organic layer was washed with water and brine, dried over sodium sulfate and concentrated *in vacuo*. The residue was chromatographed over silica gel eluted with hexane–ethyl acetate (1:1) to give 2-(trimethylsilyl)ethyl 2-(2,6-bis(benzyloxy)-4-methoxyphenylamido)ethylcarbamate (106 mg, 0.192 mmol, 70% yield).

The latter (76.4 mg, 0.139 mmol) was stirred with 20% (w/w) palladium hydroxide on carbon (10.0 mg) in methanol (2.0 ml) at room temperature for 2 h under hydrogen atmosphere. After filtration through a celite pad, the filtrate was concentrated *in vacuo*. The residue was chromatographed over silica gel eluted with hexane–ethyl acetate (2:1) to afford 2-(trimethylsilyl)ethyl 2-(2,6-dihydroxy-4-methoxyphenylamido)ethylcarbamate (45.7 mg, 0.124 mmol, 89% yield).

To a solution of the latter (45.0 mg, 0.121 mmol) in *N,N*-dimethylformamide (1.5 ml), chloromethyl methyl ether (36 μl, 0.474 mmol) and potassium carbonate (51.1 mg, 0.370 mmol) were added at room temperature. The reaction mixture was stirred for 8 h, poured into water, and extracted with ethyl acetate three times. The combined organic layer was washed with water and brine, dried over sodium sulfate, and concentrated *in vacuo*. The residue was chromatographed over silica gel eluted by hexane-ethyl acetate (2:1) to give 2-(trimethylsilyl)ethyl 2-(2-hydroxy-4-methoxy-6-(methoxymethoxy)phenylamido)ethylcarbamate (41.9 mg, 0.101 mmol, 84% yield).

To a solution of the latter (40.0 mg, 0.096 mmol) in tetrahydrofuran (THF) (1.5 ml), 1.0 M tetrabutylammonium fluoride in THF (100 µl, 0.100 mmol) was added at room temperature. The reaction mixture was stirred for 1 h at 50°C, poured into water, and extracted with ethyl acetate three times. The combined organic layer was washed with water and brine, dried over sodium sulfate, and concentrated *in vacuo*. The residue was chromatographed over silica gel eluted with chloroform–methanol (4:1) to give *N*-(2-aminoethyl)-2-hydroxy-4-methoxy-6-(methoxymethoxy)benzamide (17.2 mg, 0.063 mmol, 66% yield).

To a solution of the latter (15.0 mg, 0.056 mmol) in THF (1.5 ml), NBD-F (20.5 µl, 0.112 mmol) and *N*,*N*-diisopropylethylamine (40 μl, 0.230 mmol) were added at room temperature. The reaction mixture was stirred for 2 h, poured into water, and extracted with ethyl acetate three times. The combined organic layer was washed with water and brine, dried over sodium sulfate and concentrated *in vacuo*. The residue was chromatographed over silica gel eluted with hexane–ethyl acetate (1:1) to give 2-hydroxy-4-methoxy-6-(methoxymethoxy)-*N*-(2-(7-nitrobenzo[*c*][1,2,5]oxadiazol-4-ylamino)ethyl)benzamide (13.8 mg, 0.032 mmol, 57% yield).

The latter (9.3 mg, 0.021 mmol) was dissolved in 5% (w/v) HCl in methanol (3.0 ml) at room temperature. The solution was stirred for 5 h and concentrated *in vacuo*. The residue was dissolved in chloroform (1.5 ml), and ethanol (30 µl) and sulfuryl chloride (8.0 mg, 0.059 mmol) were added at room temperature. The reaction mixture was stirred for 2 h and concentrated *in vacuo*. The residue was chromatographed over silica gel eluted with chloroform–methanol (49:1) to give DIF-1-NBD [6.4 mg, 0.014 mmol, 65% yield (two steps)]. Analytical data for DIF-1-NBD: ^1^H NMR (600 MHz, pyridine-*d5*) δ 11.08 (1H, s), 10.59 (1H, s), 8.61 (1H, d, *J*=8.4 Hz), 6.41 (1H, d, *J*=8.4 Hz), 3.89–3.98 (4H, m), 3.91 (3H, s); ^13^C NMR (150 MHz, pyridine-*d5*) δ 169.0, 159.5, 157.6 (2C), 144.4, 139.9, 137.5, 131.5, 120.4, 105.1 (2C), 103.6, 95.5, 60.8, 42.3, 38.8; HRFABMS *m/z* 456.0085 [M-H]^+^ (456.0114 calculated for C_16_H_12_N_5_O_7_^35^Cl_2_).

### Low-magnification phase-contrast and fluorescence microscopy

Starved HM44 or Ax2 cells were incubated for the indicated times with 1.5 ml of the stalk salt solution [2 mM NaCl, 10 mM KCl, 1 mM CaCl_2_, 50 µg ml^−1^ penicillin, 100 µg ml^−1^ streptomycin sulfate and 10 mM 2-morpholinoethanesulfonic acid-KOH (MES-KOH) pH 6.2 containing various additives (DIF-1, DIF-1-BODIPY, Bu-BODIPY, DIF-1-NBD, and/or cAMP) in 35-mm tissue culture dishes (Becton Dickinson, Franklin Lakes, NJ, USA) (5×10^5^ to 10^6^ cells/dish). The cells were washed three times with the stalk salt solution and submerged in 1.5 ml of the same solution. The cells were observed at room temperature with a Leica DM IRB fluorescence microscope (Leica, Wetzlar, Germany), and digitized images were analyzed with the Leica Application Suite (version 3.3.0).

Alternatively, cells were incubated for the indicated times with additives (DIF-1 and/or cAMP) and fixed for 15–20 min at room temperature in 2 ml of 3.7% (v/v) formaldehyde in PBS(–) (20 mM phosphate buffered saline, pH 7.4), washed three times in PBS(–), stained for 30 min with DIF-1-BODIPY, Bu-BODIPY, or DIF-1-NBD, washed three times with PBS(–), and observed at room temperature under the same microscope.

### Multi-color imaging of formalin-fixed cells

Starved HM44 cells were incubated at 21°C for 1 h with 1.5 ml of the stalk salt solution containing MitoTracker (0.2 µM) in 35-mm µ-Dishes (ib81156; ibidi, Martinsried, Germany) (5×10^5^ cells/dish). Alternatively, starved HM44 cells were incubated for 20 h with 1.5 ml of the stalk salt solution containing 5 mM cAMP in 35-mm plastic dishes (5×10^5^ cells/dish) and then for 1 h with the same solution containing MitoTracker (0.2 µM). Cells were fixed for 15–20 min at room temperature with 2 ml of 3.7% (v/v) formaldehyde in PBS(–), washed three times with PBS(–), and stained for 30 min with Hoechst 33342 (1 µg ml^−1^) and DIF-1-BODIPY (5 µM) or Bu-BODIPY (5 µM). Cells were washed three times with PBS(–), submerged in 1.5 ml of PBS(–) and observed at room temperature with a Keyence BZ-9000 fluorescence microscope (Keyence, Osaka, Japan) equipped with an oil immersion 100× lens (CFI Plan Apo VC100XH) (Keyence) and multi-filters that can distinguish up to four fluorescent probes simultaneously. The original photos were deconvoluted using the Keyence BZ analyzer software to reduce ‘haze’. Z-stack sections were collected at 0.4-µm intervals, and the deconvoluted images were compiled into three-dimensional (3D) images. All color images are presented in pseudo-colors.

### *In vitro* stalk cell induction

Starved HM44 cells (2×10^5^ cells/well) were differentiated at 21°C in 12-well plates; each well was filled with 0.5 ml of stalk salt solution containing 5 mM cAMP and additives (DIF-1, DIF-2, DIF-1-BODIPY, Bu-BODIPY, DIF-1-NBD, DNP, and/or CCCP). At 48 h, the percentage of stalk cells among total cells (>150 cells/dish) was assessed by using phase-contrast microscopy.

### Small-population assay of chemotaxis

Chemotaxis toward cAMP was assessed by using Ax2 and *gbpB^–^* strains in the presence of additives as described previously (Kuwayama and Kubohara, 2009, 2016; Kuwayama et al., 2011).

### Hydrophobic index

To estimate the membrane permeability of each compound, its hydrophobic index (CP) (Fig. 1) was calculated by using ChemDraw10.0 software (Cambridgesoft, Cambridge, MA, USA).

### Statistical analysis

Unpaired Welch's *t*-test (one-tailed) was used. *P<*0.05 was considered to indicate significant differences.
